# Adipophilin and perilipin 3 positively correlate with total lipid content in human breast milk

**DOI:** 10.1038/s41598-019-57241-w

**Published:** 2020-01-15

**Authors:** Tereza Pavlova, Zdenek Spacil, Veronika Vidova, Filip Zlamal, Eliska Cechova, Zuzana Hodicka, Julie Bienertova-Vasku

**Affiliations:** 1 Research Centre for Toxic Compounds in the Environment (RECETOX), Faculty of Science, Masaryk University, Brno, Czech Republic; 2 Department of Pathological Physiology, Faculty of Medicine, Masaryk University, Brno, Czech Republic; 3 Department of Obstetrics and Gynaecology, University Hospital Brno, Brno, Czech Republic

**Keywords:** Lipids, Peptides, Membrane proteins

## Abstract

Lipids are secreted into milk as bilayer-coated structures: milk fat globules (MFGs). Adipophilin (ADRP) and perilipin 3 (TIP47) are associated with MFGs in human breast milk; however, the role of these proteins in milk lipid secretion is not fully understood. The study aimed to investigate levels of ADRP, TIP47 and total lipid content in human breast milk, their mutual correlations, and dynamics during lactation. Milk samples from 22 healthy lactating women (Caucasian, Central European) were collected at five time points during lactation (1–3, 12–14, 29–30, 88–90 and 178–180 days postpartum). Mass spectrometry-based method was used for quantification of ADRP and TIP47 in the samples. The gravimetric method was used to determine milk total lipid content. We observed distinctive trends in ADRP, TIP47 levels and lipid content in human breast milk during the first six months of lactation. We also found a significant association between lipid content and ADRP, lipid content and TIP47, and ADRP and TIP47 concentrations in breast milk at all sampling points. A mass spectrometry-based method was developed for quantifying ADRP and TIP47 in human breast milk. Strong mutual correlations were found between ADRP, TIP47 and total lipid content in human breast milk.

## Introduction

Lipids in human breast milk constitute the basic source of essential nutrients, and vitamins and polyunsaturated fatty acids, complex lipids, and bioactive components for the infant^1^. While the lipid portion forms approximately 5% of the total human milk composition, it accounts for more than 50% of the infant’s daily energy intake^2^. The exact composition of human milk lipids vary significantly during the day, season as well through different stages of lactation^2^. The same is true for the total content of lipids in the human breast milk, which is highly variably during day as well as through the whole lactation period with the average reported values of total lipid content of human milk ranging widely from 11.4 g/L to 61.8 g/L^3^

The lipids in human breast milk ensure proper neonatal growth and development, support immune system function and help synthesize bioactive lipid signalling molecules and cell membranes. While the volume of findings on milk lipid formation is growing, the number of unanswered questions is increasing at the same time^4^. A prominent research area which remains insufficiently understood is the exact molecular mechanisms of milk lipid secretion.

In mammals, milk is secreted during lactation by specialized milk-secreting epithelial cells lining the alveolar lumen of the mammary gland. The mammary gland has developed an enormous capacity to synthesize and secrete large quantities of lipids to satisfy newborns’ caloric and nutritional demands^4^. Milk lipids are synthesized on the endoplasmic reticulum of the milk-secreting cells and then accumulated in specialized intracellular organelles called cytosolic lipid droplets (CLDs). During milk lipid secretion, CLDs in milk-secreting cells move towards the apical membrane of the cells, and they are enveloped by their plasma membrane and released as structures called milk fat globules (MFGs) into the lumen of the mammary gland. In this process, the phospholipid monolayer of CLDs is encased into a bilayer of phospholipids resulting in the three-layered membrane of MFGs known as the milk fat globule membrane (MFGM). Therefore, MLGM consists of an inner phospholipid monolayer originating from CLD and an outer bilayer membrane which originates from the apical plasma membrane of the milk-secreting cells, composed mainly of cholesterol, phosphatidylcholine, and sphingomyelin^5,6^. The MFG lipid core is primarily composed of triacylglycerols (TAGs, more than 95%) and a small number of partial glycerides, phospholipids, sterols, free fatty acids, etc.^7,8^. MFGs provide important nutritional and immunological components for newborns and also regulate the rate and site of digestion in newborns’ gastrointestinal tracts^7^.

Both the surface of CLDs^9^ and MFGs^10,11^ is embedded with regulatory proteins, including the evolutionarily conserved group of proteins called perilipins. To date, CLDs lacking perilipins have not been identified in mammalian cells^12^. Evolutionarily ancient perilipins include five members with considerable sequence similarities – perilipin 1 (encoded by the PLIN1 gene), perilipin 2/ADRP (PLIN2 gene), TIP47 (PLIN3 gene), perilipin 4 (PLIN4 gene), and perilipin 5 (PLIN5 gene). A growing body of evidence suggests that the main functions of all perilipins include CLD formation and stabilization under various conditions^13^. Perilipins are either exclusively associated with CLD (perilipin 1, ADRP) or exist as exchangeable proteins stable in both the cytoplasm and CLDs (TIP47, perilipin 4 and 5)^12^. Moreover, the perilipin profiles of CLDs differ based on cell source^13^, neutral lipid composition^12^ and size^12,14^.

It has been suggested previously that the perilipins may play an essential role in milk lipid synthesis and secretion and that they are to some extent proportional to lipid content of milk^4^. Although the information on the total lipid content of milk is predominantly important in the dairy industry, it would be beneficial to have the information on the total lipid content in human milk, too, as this the total lipid content is generally an important input into formulation of infant formula and it would be beneficial to have a cheap and reliable method for establishment of total lipid content of milk in various phases of breastfeeding. Generally, the efficiency and reliability of the methods for establishment of total lipid content of milk goes in the following order: IR spectroscopy with filter technology and in mid-range of spectrum = IR spectroscopy with whole spectrum and FT in mid-range of spectrum > IR spectroscopy in the near-range spectrum ≥ turbidimetric method > ultrasound method^1^. Information about the total lipid content in breast milk is apart from the sufficient milk lipid production needed for proper neonatal growth and develoment necessary to normalize levels of non-polar organic pollutants in human breast milk such as persistent pesticides or polychlorinated biphenyls in analysis, hence is of a great importance for current toxicology.

The study therefore aims i) to establish a quantitative protein assay for selected perilipins in human breast milk (perilipin 1, ADRP, TIP47 and perilipin 5) to investigate the levels of these proteins in breast milk during the first 180 days after the birth, ii) to examine the correlation between levels of perilipin 1, ADRP, TIP47 and perilipin 5 and total lipid content in human breast milk and iii) to investigate possible role of investigated perilipins for the prediction of the total lipid content.

## Methods

### Study subjects and design

A cohort of 22 healthy lactating women (Caucasian, Central-European) was recruited between October 2009 and June 2010. All women completed a demographic questionnaire. Signed informed consent forms were obtained from all participants and archived. The study was approved by the Committee for Ethics of Medical Experiments on Human subjects, Faculty of Medicine of Masaryk University (Brno, Czech Republic) under the Declaration of Helsinki. The methods used in the study and described below were carried out in accordance with the relevant guidelines and regulations. The authors confirm that the data supporting the findings of this study are available within the article and in supplementary materials.

Inclusion criteria to the study were 1) spontaneous conception, 2) uneventful, singleton pregnancy, 3) spontaneous, uncomplicated delivery, 4) a normal oral 75-g oral glucose tolerance test between 24 and 28 weeks of gestation based on World Health Organization (WHO) criteria^15^, 5) appropriate-for-gestational-age neonate, 6) no pre-pregnancy or pregnancy hormonal therapy, previous fertility treatment, surgical or induced delivery, 7) pre-conception body mass index under 35 kg/m^2^ and 8) primiparity.

Milk samples were collected by manual expression from both breasts always 2 hours after breastfeeding or after a previous manual milk expression. This approach was based on the study by Lai *et al*. who report that the 2^nd^ to 7^th^ hour after breastfeeding provides a relatively precise estimate of the volume of the milk produced^16^. Also, this approach is generally approved in most of the milk banks^17^. From the study participants, a total of 5 ml of milk was collected at the following time points: day 1–3 (time point 1), 12–14 (2), 28–30 (3), 88–90 (4) and 178–180 (5) after birth, i.e. 5 times within the 180 days of the study. The obtained samples were vortexed, distributed into 10 mL and 200 µl aliquots and kept frozen at −80 °C until further sample processing for analysis.

### Chemicals and solvents

Isotopically labeled proteotypic peptides (SpikeTides^TM^ L and Peptides SpikeTides^TM^ TQL) were purchased from JPT Peptide Technologies (Germany) and used to detect and quantify selected perilipins. MS grade Trypsin Gold (Promega) was used for enzymatic protein digestion. Solid phase extraction (SPE) was performed to desalt and purify peptides using Bond Elut C18 200 mg (Agilent). Ammonium bicarbonate buffer (200 mmol/L, pH = 8, Sigma Aldrich) was added to breast milk samples to adjust pH for tryptic digestion; LC-MS grade formic acid (FA, Sigma Aldrich) was added after digestion to acidify samples for loading on SPE and 0.1% FA was also used as an ultra-high performance liquid chromatography (UHPLC) mobile phase additive. Organic solvents for mobile phase, sample processing and dilution of stock solutions, i.e. liquid chromatography and mass spectrometry (LC-MS) grade methanol and acetonitrile were purchased from Avantor. Ammonia (Lach-Ner), ethanol (Merck), diethyl ether (Promochem) and n-hexane (Lach-Ner) were used for lipid extraction. Ultrapure water (resistivity of 18.2 MΩ.cm at 25 °C) was obtained using the Millipore purification system (Simplicity 185 system, Millipore Corp.).

### Total lipid content of milk

The liquid-liquid extraction Röse–Gottlieb reference method used to determine the total lipid content of milk^18^ was modified to accommodate lower sample amounts compared to the original method protocol. Milk samples aliquots (10 mL) were refrigerated overnight to thaw and then sonicated for 20 min. 5 mL of each milk sample was mixed with 750 µL of ammonia in water (25%), 5 mL of ethanol, 12.5 mL of diethyl ether and 12.5 mL of n-hexane and shaken vigorously once each solvent was added. Samples were centrifuged, and the upper phase was collected into a weighed vial. Diethyl-ether and n-hexane were then added again to the lower phase, mixed and centrifuged; the collected upper layer (i.e. the second fraction) was combined with the first fraction. Finally, a third fraction was collected in the same manner. The solvent was evaporated from the combined fraction under a stream of nitrogen and the total lipid weight was gravimetrically determined.

### Protein concentrations in milk

#### Protein assay design

Crude stable-isotopically labelled proteotypic peptides (SpikeTides^TM^ L) (Table 1) were selected for four perilipins (perilipin 1, ADRP, TIP47 and perilipin 5) according to the UniProt database (www.uniprot.org) and SRMAtlas repository (www.srmatlas.org) and used to generate a transition list for selected reaction monitoring (SRM) as previously described^19^. Stable-isotopically labeled peptides with trypsin cleavable tags (SpikeTides^TM^ TQL) of certified exact concentrations were used for the absolute quantification of ADRP and TIP47 in breast milk.

**Table 1:** Crude stable-isotopically labeled proteotypic peptides for perilipins.

Protein	Gene	Peptide sequence	SIL peptideSpikeTides^TM^
ADRP	PLIN2	SELLVEQYLPLTEEELEK	L and TQL
ADRP	PLIN2	TITSVAMTSALPIIQK	L
ADRP	PLIN2	SQQTISQLHSTVHLIEFAR	L
ADRP	PLIN2	DSVASTITGVMDK	L and TQL
ADRP	PLIN2	EVSDSLLTSSK	L and TQL
Perilipin 1	PLIN1	LASGGADLALGSIEK	L
Perilipin 1	PLIN1	VLHLTPAPAVSSTK	L
TIP47	PLIN3	IATSLDGFDVASVQQQR	L and TQL
TIP47	PLIN3	DTVATQLSEAVDATR	TQL
Perilipin 5	PLIN5	SVSHAVDVVLEK	L
Perilipin 5	PLIN5	SVDALQTAFADAR	L

The listed SpikeTides^TM^ L peptides (L) were used to screen for four perilipins in breast milk and to generate a transition list. Perilipins with concentrations above the detection limit in breast milk were quantified in milk using stable-isotopically labeled (SIL) peptides with trypsin cleavable tags (TQL) with the same sequence as SpikeTides^TM^ L.

#### Quantitative method optimization

Perilipin concentrations in individual samples were measured and mean concentrations at specific time points were calculated. The pooled milk samples were prepared by mixing 10 µL of milk from each individual sample and subsequently used for the preparation of matrix-matched calibration curve (MM). MM calibration was prepared using pooled sample and a mix of TQL peptides at six concentration levels within a range of 5–400 nM (Supplemental Fig. 1, Table 2). Limit of detection (LOD) and limit of quantification (LOQ) values were calculated from the MM calibration curve using the lowest concentration level with a coefficient of variance < 20% (n = 6) to determine standard deviation (SD), e.g. 3*SD divided by SLOPE (for LOD) and 10*SD divided by SLOPE (for LOQ)^19^. Linearity within the 5–400 nM concentration range was R^2^ > 0.99.

**Table 2:** Calibration parameters in matrix-matched solution.

Protein	Peptide	LOD (nM)	LOQ (nM)	Linear range (nM)	Slope	Intercept	R^2^	R^2^ norm
ADRP	DSVASTITGVMDK	1.6	4.8	5–400	122.8	633.9	0.977	0.994
ADRP	EVSDSLLTSSK	1.1	3.2	5–400	261.8	1731.4	0.992	1.000
TIP47	DTVATQLSEAVDATR	1.6	4.8	5–400	83.9	818.1	0.980	0.997

LOD, the limit of detection; LOQ, the limit of quantification; R^2^, Pearson Coefficient of Determination; R^2^ norm, Coefficient of Determination after adjusting for native peptide.

Enzymatic digestion duration (8, 16 and 24 h) was optimized, and 16 h trypsin digestion was determined as optimal based on the highest response of light and heavy peptides compared to the remaining reaction times (Supplemental Fig. 2).

#### Sample preparation

Milk samples aliquots (200 µL) were refrigerated overnight to thaw and then sonicated for 5 min. 10 µL of ammonium bicarbonate buffer (200 mM, pH = 8), 10 µL of TQL peptides standard solution and 3 µL of trypsin (1 µg/µL) were added to 30 µL of breast milk, followed by incubation at 37 °C. Individual TQL peptide concentrations in the standard solution were as follows: SELLVEQYLPLTEEELK (ADRP), 1200 nmol/L; DSVASTITGVMDK (ADRP), 750 nmol/L; EVSDSLLTSS (ADRP), 750 nmol/L; DTVATQLSEAVDATR (TIP47), 300 nmol/L; IATSLDGFDVASVQQQR, 150 nmol/L. Trypsin digestion was terminated after 16 hours by adding 300 µL of 2% FA in water. SPE cartridges were conditioned as follows: 1 mL of methanol, 1 mL of 50% acetonitrile (with 2% FA) and 1 mL of 2% FA. The sample was loaded into a cartridge, washed with 2 mL of 2% FA and eluted with 1 mL of 50% acetonitrile (with 2% FA). The sample solvent was removed under a stream of nitrogen, and the sample was re-dissolved in 30 µL of 5% acetonitrile, with final peptide concentrations as follows: 400, 250, 250, 100 and 50 nmol/L, respectively.

#### Protein quantification – UHLPC-MS/MS (SRM)

Samples were analyzed using UHPLC and tandem mass spectrometry (MS/MS), specifically by SRM. The UHPLC system (1290 Infinity II) and triple quadrupole mass analyzer (model 6495) were utilized (Agilent Technologies, CA, USA). Reversed-phase UHPLC separation utilized analytical column C18 CSH column (1.7 µm, 2.1 × 100 mm, Waters). MS/MS conditions were optimized using Optimizer software (Agilent Technologies). UHPLC separation was performed with water (A) and acetonitrile (B), both with an addition of 0.1% FA at 0.3 mL/min using 35 min analytical gradient (0.00 min 5% B, 25.00 min 30% B, 25.30 min 95% B, 30.00 min 95% B, 31.00 min 5% B, 35.00 min 5% B). Milk samples were analyzed in positive ion and dynamic SRM mode with a 3.5 min retention time window. The assay library is specified in supplementary materials (Supplemental Table 1). Five SRM transitions were used as qualifiers, and the most intense signal was used as a quantifier. The quantifiers are marked in bold in Supplemental Table 1.

### Statistical analyses

All statistical analyses were carried out using statistical software R (version 3.3.3). Conventional values of *p* < 0.05 were considered statistically significant. Descriptive statistics of variables were characterized by mean, median, standard deviation (SD), minimum and maximum values. The characteristics in the figures are depicted as means ± SDs.

The differences of ADRP/TIP47/total lipid concentrations between time points were tested via linear regression mixed effects model for the natural logarithm of the variables with time as fixed effect and subject as a random effect. The final p-values were corrected using Benjamini-Hochberg-Yekuteli method. Moreover, Helmert contrasts were used to compare TIP47 levels between the time points. Helmert contrasts were performed in linear regression mixed effects model for the natural logarithm of TIP47 (as an independent variable) and with time as fixed effect and subject as a random effect. The contrast is a way how to compare mean values between the combination of levels in categorical variables. Helmert contrasts compare given level with a combination of preceding levels.

Pearson (r) and partial correlation (r_par_) coefficients were used to quantify the strength of mutual relationships between ADRP, TIP47 and total lipid concentrations (logarithmically transformed) in each time point. R measures the power of the relationship between a pair of variables with no relation to other variables. On the other hand, r_par_ allows for adjusting the connection between the two variables across the resting variables in the dataset. For example, in the set of three variables, we find the Pearson correlation coefficient is significant; however, r_par_ is no longer meaningful. This fact can be interpreted as the relation between the two variables can be explained by the effect of the third variable, thereby the third variable plays the role of a confounder. R_par_ was calculated using this formula:

$${r}_{yx,z}=\frac{{r}_{yx}-{r}_{yz}{r}_{xz}}{\sqrt{1-{r}_{yz}^{2}}\sqrt{1-{r}_{xz}^{2}}}$$

After calculating the Pearson correlation coefficients and their significance, the p-values were corrected using Benjamini-Hochberg-Yekuteli method. This method belongs to the False Discovery Rate procedure which allows controlling for the number of false positives by controlling the false detection rate. Moreover, this method also takes into account the assumption of positive dependence in multiple testing as expected in time dependence data.

The correlation structure was also studied by means of principal component analysis (PCA). PCA is one of the multivariate statistical methods enabling to uncover correlation structure within a set of variables. In our study, PCA was performed to describe the correlation structure of the three variables, i.e., ADRP, TIP47, and total lipid concentrations, in all-time points using correlation matrix.

## Results

### Protein quantification

The calibration curve was prepared in the pooled breast milk sample using mixture contained TQL peptides corresponding to ADRP and TIP47 proteins. The calibration curve was then normalized to native peptides present in breast milk samples. Based on that, LOD and LOQ of peptides were determined and are listed in Table 2. Protein concentrations were below LOD in 4 and 5 from 85 samples for ADRP and TIP47, respectively. The ADRP concentrations range from 55.3 to 541.4 ng/mL and TIP47 from 11.3 to 174.7 ng/mL.

ADRP was quantified using two TQL peptides, DSVASTITGVMDK and EVSDSLLTSSK (Table 2, Supplemental Fig. 3). These peptides were selected based on their similar concentrations in the analyzed samples (Pearson correlation coefficient = 0.095, *p* < 0.001). Mean concentration of the two native peptides quantified in the sample was determined as final. TIP47 was quantified using one TQL peptide, DTVATQLSEAVDATR (Table 2). The second TQL peptide (IATSLDGFDVASVQQQR) was under LOD both in the pooled sample and in all breast milk samples.

### ADRP and TIP47 concentrations and total lipid content in breast milk

**Figure 1:**
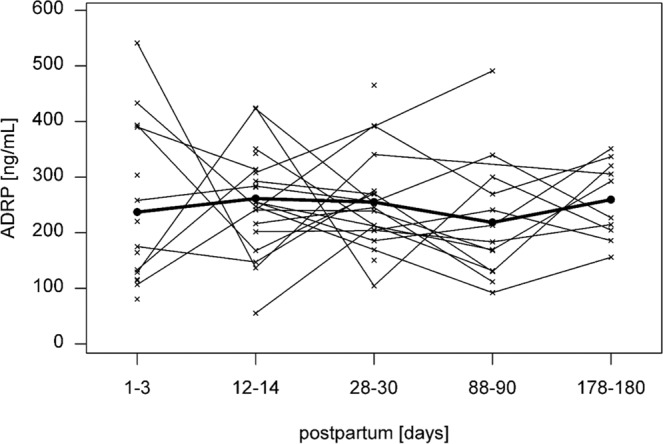
Levels and a trend of ADRP in human breast milk during lactation. Mean ± SD of the concentrations of ADRP in breast milk at 1–3, 12–14, 28–30, 88–90 and 178–180 days postpartum. The crosses represent individual milk samples.

**Figure 2:**
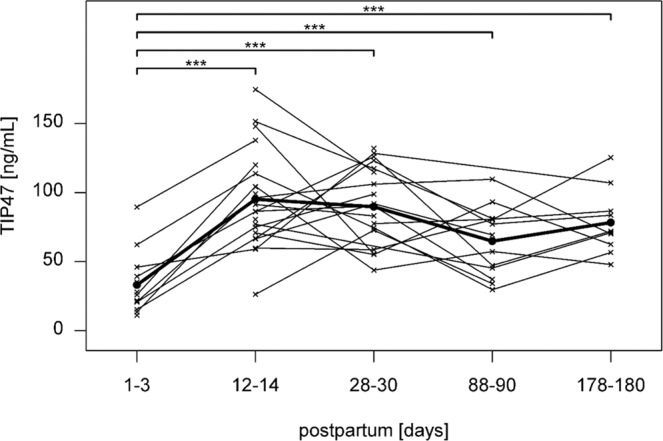
Levels and a trend of TIP47 in human breast milk during lactation. Mean ± SD of the concentrations of TIP47 in breast milk at day 1–3, 12–14, 28–30, 88–90 and 178–180 days postpartum. The crosses represent individual milk samples. ***p-value less than 0.001.

**Figure 3:**
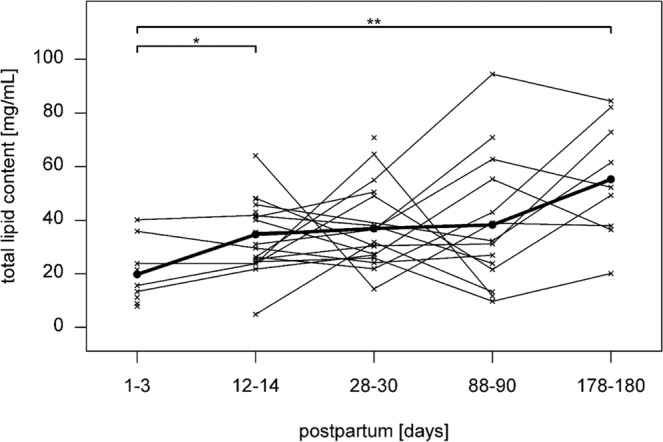
Levels and a trend of the total lipid content in human breast milk during lactation. Mean ± SD of the concentrations of the total lipid content in breast milk at day 1–3, 12–14, 28–30, 88–90 and 178–180 days postpartum. The crosses represent individual milk samples. *p-value between 0.01 and 0.05. **p-value between 0.001 and 0.01.

The baseline characteristics of the study subjects are summarized in Table 3. ADRP (Fig. 1) and TIP47 levels (Fig. 2) and total lipid content (Fig. 3) were measured in each individual sample at each time point. We found no significant differences between ADRP breast milk concentrations at different time points during lactation. However, TIP47 concentration in breast milk was found to be significantly increased at all time points compared to time point 1 (*p* < 0.001). Compared to time point 1, the total lipid content of milk was significantly increased at time point 2 (*p* = 0.035) and at time point 5 (*p* = 0.003). The p-values were corrected using Benjamini-Hochberg-Yekuteli method, as described in the Statistical analyses section.

**Table 3:** Anthropometric data of study subjects.

	Mean	SD	Median	Min	Max
Maternal age [years]	29.9	3.3	30.4	25.6	36.6
Preconception BMI [kg/m^2^]	22.8	4.4	21.6	17.7	33.4
BMI at delivery [kg/m^2^]	27.7	4.7	26.9	22.0	38.9
Pregnancy weight gain [kg]	15.2	4.2	15.0	8.0	25.0
Birth length [cm]	49.9	1.6	50.0	47.0	52.0
Birth weight [kg]	3.2	0.6	3.3	2.2	4.5

Using Helmert contrasts we found the mean difference between time point 1 and time points 2–5 to be 0.90 (95% CI: 0.68;1.12; *p* < 0.001), transformed back to the original scale resulting with 147% increase in mean values in time point 1 compared to time points 2–5 (95% CI: 98;308%). We also found borderline significant decrease of TIP47 (after p-value adjustment) between time points 2 and 4 of value −0.37 (95% CI: −0.65;−0.08, *p* = 0.014, p_adj = _0.082) on logarithmic scale corresponding to 31% decrease (95% CI: 7;48%) on original scale. We also found borderline significant decrease of TIP47 (after p-value adjustment) between time points 3 and 4 of value −0.34 (95% CI: −0.62;−0.06, *p* = 0.020, p^adj = ^0.098) on logarithmic scale corresponding to 29% decrease (95% CI: 5;46%) on original scale. The differences between the rest timepoints (2 vs. 3, 2 vs. 5, 3 vs. 5 and 4 vs. 5) are not significant.

### Correlation

We found significant correlations between total lipid content and ADRP, between lipid content and TIP47 and also between ADRP and TIP47 at all lactation time points. The only exception is the relationship between the lipid content of milk and TIP47 concentration 1–3 days postpartum where the correlation was found to be not significant (Table 4). The p-values were corrected using Benjamini-Hochberg-Yekuteli method, as described in the Statistical analyses section.

**Table 4:** Correlations between ADRP, TIP47 and lipid content of human breast milk at different time points during lactation.

Time point	Lipid content vs. ADRP				Lipid content vs. TIP47					ADRP vs. TIP47						
	n	r	95% CI	p(r)	r_par_	p(r_par_)	r	p(r)	95% CI	r_par_	p(r_par_)	r	95% CI	p(r)	r_par_	p(r_par_)
1–3	5	0.83	(0.24; 0.97)	**0.038**	0.74	0.591	0.64	0.244	(−0.18;0.93)	−0.39	1.000	0.89	(0.46;0.98)	**0.014**	0.84	0.451
12–14	20	0.74	(0.46; 0.87)	**< 0.001**	0.60	**0.040**	0.53	**0.030**	(0.14;0.77)	0.02	1.000	0.71	(0.41;0.87)	**0.001**	0.55	0.120
28–30	18	0.88	(0.72; 0.95)	**< 0.001**	0.62	**0.040**	0.8	**< 0.001**	(0.56;0.92)	0.17	1.000	0.86	(0.68;0.94)	**< 0.001**	0.55	0.120
88–90	13	0.72	(0.33; 0.90)	**0.007**	0.36	0.591	0.71	**0.010**	(0.32;0.89)	0.33	1.000	0.8	(0.49;0.93)	**0.001**	0.59	0.167
178–180	9	0.8	(0.40; 0.94)	**0.007**	0.53	0.591	0.82	**0.008**	(0.45;0.95)	0.59	1.000	0.72	(0.23;0.92)	**0.023**	0.19	1.000

n, number of mothers sampled at a given time point; r, Pearson correlation coefficient; CI, confidence interval for Pearson correlation coefficient; r_par_, partial correlation coefficient, i.e. Pearson correlation coefficient following the removal of the effects of the third variable; time point 1–3, 12–14, 28–30, 88–90, 178–180 days postpartum. P-values are presented for r (p(r)) and r_par_ (p(r_par_)) and are corrected using Benjamini-Hochberg-Yekuteli method.

The correlation was visualized using PCA (Supplemental Fig. 4). The first principal component includes in different time points 77–90% variability, suggesting strong mutual association between all three variables. Specifically, during 1–3 days postpartum the proportion of the variance associated with the first principal component (PC1) is 0.86, second principal component (PC2) is 0.12 and third principal component (PC3) is 0.02. During 12–14 days postpartum PC1 = 0.77, PC2 = 0.16, PC3 = 0.07; 28–30 days postpartum PC1 = 0.90, PC2 = 0.07, PC3 = 0.03, 88–90 days postpartum PC1 = 0.83, PC2 = 0.10, PC3 = 0.07 and 178–180 days postpartum PC1 = 0.86, PC2 = 0.09 and PC3 = 0.05.

### Confounding factors

When comparing r and r_par_ within the three relationships, i.e., lipid content vs. ADRP, lipid content vs. TIP47 and ADRP vs. TIP47 (Table 4), the change of r after correction (r_par_) are the greatest in the relationship lipid content vs. TIP47. In other words, when we remove the effect of ADRP in the relationship between TIP47 and the lipid content, there will be the most significant decrease in the correlation between TIP47 and lipid content. In addition, the values of correlation between the lipid content and ADRP are still relatively high even after correction for TIP47, especially in the first three time points. These facts suggest that ADRP could play the role of a confounding factor in the relationship between TIP47 and the lipid content of milk at least within the first 90 days postpartum.

## Discussion

This study investigated selected perilipins involved in the process of milk lipid secretion, to contribute to the current understanding of molecular frameworks regulating milk lipid synthesis and secretion. We initially decided to choose specific peptide standards (proteotypic peptides) representing four perilipins (perilipin 1, ADRP, TIP47 and perilipin 5). We screened these four perilipin proteins in human breast milk, detecting ADRP and TIP47 in concentrations above LOD. We did not identify perilipin 1 and 5. While perilipin 1 is presumably expressed in the mammary gland and localized exclusively in CLDs^20^, it was previously not detected in milk human breast milk^21^. Perilipin 5 has never been associated with MFGs or breast milk. If trace amounts of perilipin 1 and 5 are indeed present in human breast milk, their concentrations were below the detection limit of the described SRM protein assay. This study therefore further focused on quantifying ADRP and TIP47 in human breast milk and investigating their relation to the lipid content of breast milk.

### Adipophilin

ADRP is among the most abundant proteins found in both CLDs and MFGs. It has been hypothesized to be a key player in milk lipid formation and secretion^22^. ADRP was initially identified in the teratoma-derived adipogenic cell line (1246 Cells) as an mRNA molecule expressed early during adipocyte differentiation^23^. The 50 kDa protein was thus named adipose differentiation-related protein (ADRP)^24^. ADRP expression is not limited to adipocytes but is ubiquitous, in contrast to perilipins 1, 4 and 5, which exhibit more limited tissue expression^12^. ADRP transcript levels in milk-secreting cells are significantly higher than in other cell types, including adipocytes. Overall, it seems that the increased expression of ADRP is unique to milk-secreting cells^21^. Although ADRP levels have been reported to correlate with lipid accumulation in a variety of cells and tissues^24–26^, to the best of our knowledge, this is the first study to show a correlation between ADRP and the lipid content of human breast milk.

Only one previous study reports on the quantification of ADRP in human breast milk^27^ utilizing the immunoanalytical ELISA technique. In this study, ADRP was quantified in human breast milk during a 6-month lactation period. ADRP concentrations measured by ELISA were approximately one order of magnitude higher compared to our results measured by SRM protein assay. Specifically, at time point 1 we measured mean protein concentrations of 0.24 ± 0.14 µg/mL compared to 1.98 ± 0.12 µg/mL determined by Mitrova *et al*.^27^, at time point 3 0.26 ± 0.09 µg/mL vs. 2.83 ± 0.21 µg/mL, at time point 4 0.25 ± 0.17 µg/mL vs. 2.39 ± 0.17 µg/mL and at time point 5 0.26 ± 0.07 ng/mL vs. 2.57 ± 0.16 µg/mL. The disparity may be due to the different sample preparation, as Mitrova *et al*. used skimmed milk in comparison to whole milk utilized in the present study, or different quantification methods, i.e., ELISA vs. SRM determination by tandem mass spectrometry.

### Perilipin 3

Like ADRP, TIP47 is also expressed in the mammary gland^20^ and has been associated with MFGs in human breast milk^10^. TIP47 was initially identified as a 48 kDa placental protein PP17^28^. In contrast to ADRP, the association of TIP47 with CLDs is controversial. While TIP47 has been reported to be associated with intracellular CLDs^10,29,30^, it is not associated with CLDs in milk-secreting cells but is diffusely distributed in the milk-secreting cell cytoplasm; furthermore, its expression patterns are not correlated with CLD accumulation^20^. TIP47 has been detected in a fat-depleted fraction but not in MFGs in mouse milk; its expression has been found to decrease during mammary gland development^21^. On the other hand, TIP47 has been detected in human breast milk MFGs^10,31^. Thus, both the localization and the biological role of TIP47 in breast milk remain unclear. By comparison, ADRP in secretory epithelial cells is associated exclusively with CLDs.

The different localization of ADRP and TIP47 in secretory cells is consistent with our results, which indicate a different relationship of these perilipins to the lipid content of milk. ADRP looks like a confounder in the relationship between ADRP, TIP47, and lipid content and we suggest that the correlation between TIP47 and total lipid content is dependent on the association between ADRP and TIP47. Besides, the dynamics of the two perilipins seems different throughout the lactation period. We observed a constant ADRP level between days 1–180 of lactation, while TIP47 level significantly increases between time points 1–3 and 12–14 and then the level remains constant during the rest of observed lactation period.

### Lipid content of milk

Information about the total lipid content in breast milk is, e.g. concerning sufficient milk lipid production needed for proper neonatal growth and development or to normalize levels of non-polar organic pollutants in human breast milk such as persistent pesticides or polychlorinated biphenyls^32^. However, the original referenced method for the determination of the lipid content of breast milk required a relatively large volume of breast milk and the use of highly flammable solvents; moreover, the process was difficult, time-consuming and highly prone to error^18^. On the other hand, the correlation between ADRP and the lipid content of breast milk presented in our study brings an alternative way of determining the total lipid content of breast milk based on ADRP concentration. Nevertheless, the method has to be validated.

Our hypothesis about the correlation between the lipid content of milk and ADRP is supported by the fact that ADRP colocalizes with CLDs secreted by mammary secretory epithelial cells into milk^21^. Furthermore, it is also one of the most abundant proteins found in MFGs^11^. In this study, we have discovered the correlation between the lipid content of milk and ADRP levels and thus confirmed the hypothesis. The finding suggests that ADPR levels at specific time points during lactation are predictive of the total lipid content in breast milk at investigated time points within the lactation period.

### Strengths and weaknesses of the study

To the best of our knowledge, this is the first study to show a correlation between ADRP as well as TIP47 and the lipid content of human breast milk. This provides a possible alternative way for milk lipid content determination. An indisputable advantage of this way is a small amount of sample, i.e., 30 µL of breast milk. The method would be less time-consuming and prone to error compared to the reference method^18^. Nevertheless, the technique requires the UHPLC system and mass analyzer together with specific TQL peptides for protein quantification. Another advantage of the study is the absolute sequence specificity of SRM determination by tandem mass spectrometry. It has to be mentioned, too, that the main weakness of the study is the reproducibility of the MS method as the method has not been validated yet using the recombinant protein. Moreover, another potential interpretation bias could stem from the fact that detected perilipins could originate from the mammary epithelial cells in the breast milk, which we did not consider in the design of our experiment.

## Conclusion

We have established a UHPLC-MS/MS assay for the absolute quantification of ADRP and TIP47 in human breast milk. We found strong correlations between ADRP and lipid content, TIP47 and lipid content and also between ADRP and TIP47 in human breast milk.

## Supplementary information

Supplementary Info.
