# Supplementary material for: Adipophilin and perilipin 3 positively correlate with total lipid content in human breast milk
Source: Sci Rep. 2020 Jan 15;10:360. doi: 10.1038/s41598-019-57241-w (PMC6962152; doi:10.1038/s41598-019-57241-w)
Supplement: Supplementary file 1 — Supplementary Info. [file 41598_2019_57241_MOESM1_ESM.doc]

**Supplementary Material for**

# Adipophilin and perilipin 3 positively correlate with total lipid content in human breast milk

Tereza Pavlova^1,2,*^, Zdenek Spacil^1^, Veronika Vidova^1^, Filip Zlamal^1,2^, Eliska Cechova^1^, Zuzana Hodicka^3^, Julie Bienertova-Vasku^1,2^

*^1^Research Centre for Toxic Compounds in the Environment (RECETOX), Masaryk University, Czech Republic*

*^2^ Department of Pathological Physiology, Masaryk University, Faculty of Medicine, Brno, Czech Republic*

*^3^Department of Obstetrics and Gynaecology, University Hospital Brno, Brno, Czech Republic*

**Supplemental Table 1: SRM assay library for positive ion detection mode**

| **Peptide Name** | **ISTD^1^** | **Precursor Ion** | **Product Ion** | **ColisionEnergy(V)** | **RetentionTime (min)** |
| --- | --- | --- | --- | --- | --- |
| **DSVASTITGVMDK.heavy** | **Yes** | **666.3** | **1030.5** | **26.2** | **13.8** |
| DSVASTITGVMDK.heavy | Yes | 666.3 | 959.5 | 26.2 | 13.8 |
| DSVASTITGVMDK.heavy | Yes | 666.3 | 658.3 | 26.2 | 13.8 |
| DSVASTITGVMDK.heavy | Yes | 666.3 | 302.1 | 26.2 | 13.8 |
| DSVASTITGVMDK.heavy | Yes | 666.3 | 203.1 | 26.2 | 13.8 |
| **DSVASTITGVMDK.light** | **No** | **662.3** | **1022.5** | **26.2** | **13.8** |
| DSVASTITGVMDK.light | No | 662.3 | 951.5 | 26.2 | 13.8 |
| DSVASTITGVMDK.light | No | 662.3 | 650.3 | 26.2 | 13.8 |
| DSVASTITGVMDK.light | No | 662.3 | 302.1 | 26.2 | 13.8 |
| DSVASTITGVMDK.light | No | 662.3 | 203.1 | 26.2 | 13.8 |
| **DTVATQLSEAVDATR.heavy** | **Yes** | **793.9** | **971.5** | **29.8** | **14** |
| DTVATQLSEAVDATR.heavy | Yes | 793.9 | 858.4 | 29.8 | 14 |
| DTVATQLSEAVDATR.heavy | Yes | 793.9 | 642.4 | 29.8 | 14 |
| DTVATQLSEAVDATR.heavy | Yes | 793.9 | 472.2 | 29.8 | 14 |
| DTVATQLSEAVDATR.heavy | Yes | 793.9 | 217.1 | 29.8 | 14 |
| **DTVATQLSEAVDATR.light** | **No** | **788.9** | **961.5** | **29.8** | **14** |
| DTVATQLSEAVDATR.light | No | 788.9 | 848.4 | 29.8 | 14 |
| DTVATQLSEAVDATR.light | No | 788.9 | 632.3 | 29.8 | 14 |
| DTVATQLSEAVDATR.light | No | 788.9 | 462.2 | 29.8 | 14 |
| DTVATQLSEAVDATR.light | No | 788.9 | 217.1 | 29.8 | 14 |
| **EVSDSLLTSSK.heavy** | **Yes** | **587.3** | **945.5** | **23.9** | **7.3** |
| EVSDSLLTSSK.heavy | Yes | 587.3 | 543.3 | 23.9 | 7.3 |
| EVSDSLLTSSK.heavy | Yes | 587.3 | 430.2 | 23.9 | 7.3 |
| EVSDSLLTSSK.heavy | Yes | 587.3 | 329.2 | 23.9 | 7.3 |
| EVSDSLLTSSK.heavy | Yes | 587.3 | 242.2 | 23.9 | 7.3 |
| **EVSDSLLTSSK.light** | **No** | **583.3** | **937.5** | **23.9** | **7.3** |
| EVSDSLLTSSK.light | No | 583.3 | 535.3 | 23.9 | 7.3 |
| EVSDSLLTSSK.light | No | 583.3 | 422.2 | 23.9 | 7.3 |
| EVSDSLLTSSK.light | No | 583.3 | 321.2 | 23.9 | 7.3 |
| EVSDSLLTSSK.light | No | 583.3 | 234.1 | 23.9 | 7.3 |
| **IATSLDGFDVASVQQQR.heavy** | **Yes** | **923** | **1359.7** | **33.8** | **14.1** |
| IATSLDGFDVASVQQQR.heavy | Yes | 923 | 1040.5 | 33.8 | 14.1 |
| IATSLDGFDVASVQQQR.heavy | Yes | 923 | 826.4 | 33.8 | 14.1 |
| IATSLDGFDVASVQQQR.heavy | Yes | 923 | 755.4 | 33.8 | 14.1 |
| IATSLDGFDVASVQQQR.heavy | Yes | 923 | 569.3 | 33.8 | 14.1 |
| **IATSLDGFDVASVQQQR.light** | **No** | **918** | **1349.6** | **33.8** | **14.1** |
| IATSLDGFDVASVQQQR.light | No | 918 | 1030.5 | 33.8 | 14.1 |
| IATSLDGFDVASVQQQR.light | No | 918 | 816.4 | 33.8 | 14.1 |
| IATSLDGFDVASVQQQR.light | No | 918 | 745.4 | 33.8 | 14.1 |
| IATSLDGFDVASVQQQR.light | No | 918 | 559.3 | 33.8 | 14.1 |
| **SELLVEQYLPLTEEELEK.heavy** | **Yes** | **724** | **1095.6** | **21.3** | **21.7** |
| SELLVEQYLPLTEEELEK.heavy | Yes | 724 | 962.5 | 21.3 | 21.7 |
| SELLVEQYLPLTEEELEK.heavy | Yes | 724 | 885.4 | 21.3 | 21.7 |
| SELLVEQYLPLTEEELEK.heavy | Yes | 724 | 548.3 | 21.3 | 21.7 |
| SELLVEQYLPLTEEELEK.heavy | Yes | 724 | 443.3 | 21.3 | 21.7 |
| **SELLVEQYLPLTEEELEK.light** | **No** | **721.4** | **1087.6** | **21.3** | **21.7** |
| SELLVEQYLPLTEEELEK.light | No | 721.4 | 962.5 | 21.3 | 21.7 |
| SELLVEQYLPLTEEELEK.light | No | 721.4 | 877.4 | 21.3 | 21.7 |
| SELLVEQYLPLTEEELEK.light | No | 721.4 | 544.3 | 21.3 | 21.7 |
| SELLVEQYLPLTEEELEK.light | No | 721.4 | 443.3 | 21.3 | 21.7 |

*Additional instrument settings for Agilent 6495 QqQ were positive polarity,* *fragmentor = 380 V, cell accelerator voltage = 5 V and retention time window = 3.5 min. The quantification SRM transitions are in bold.*

*^1^Internal standard*

**Supplemental Figure 1: Matrix matched calibration curves**

**
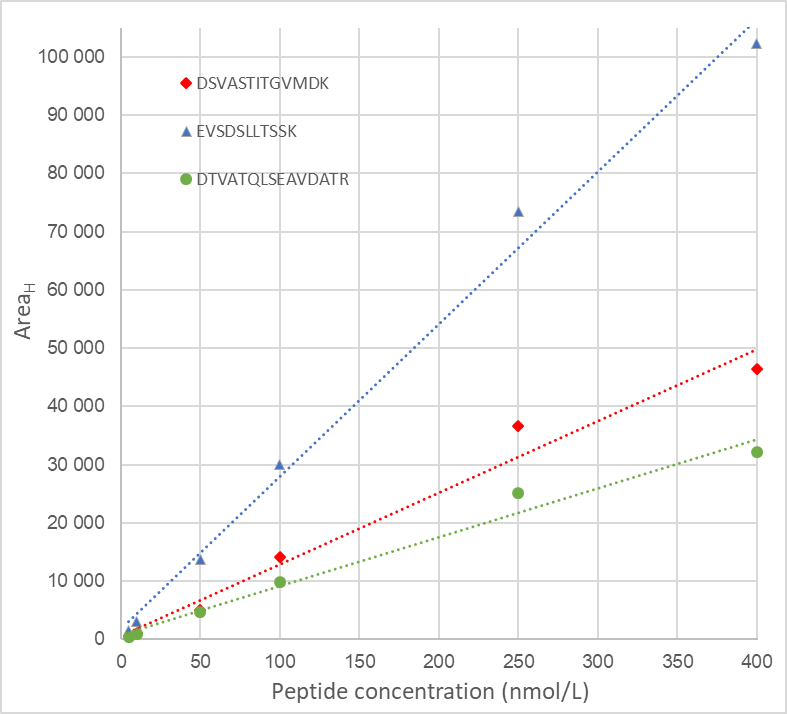

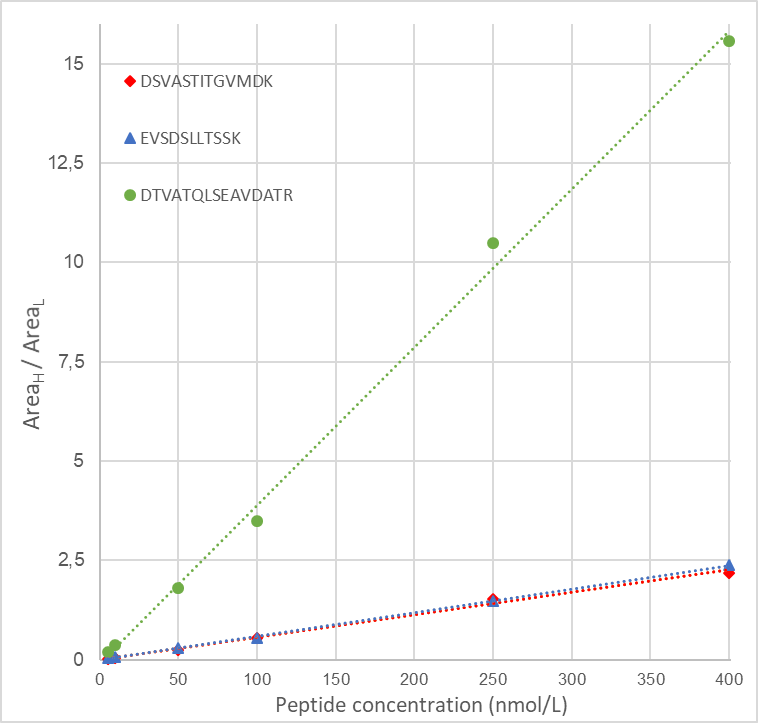
**

*The correlation between standard peptides concentrations in the breast milk and their responses. Calibration curves of TQL peptides after trypsin digestion (heavy standard) (1) and after normalization on native peptide (light) (2).*


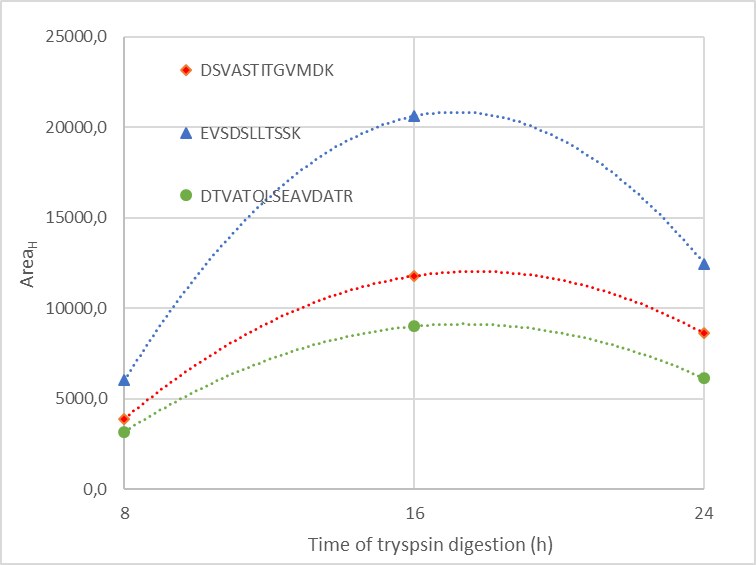

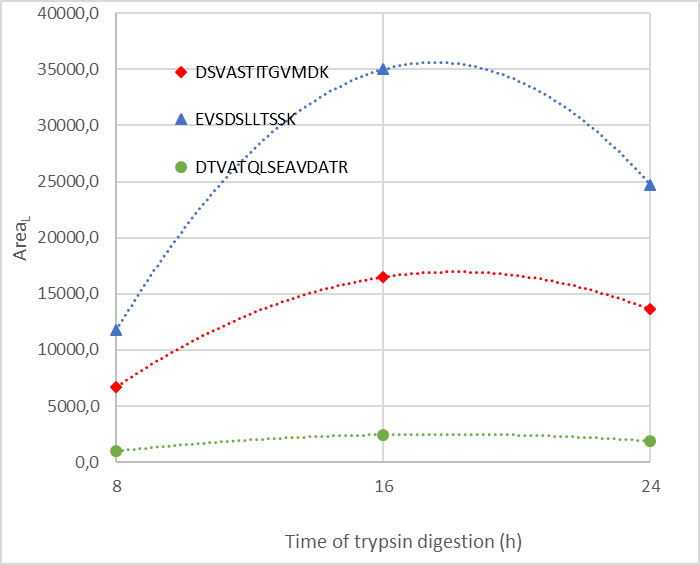
**Supplemental Figure 2: Trypsin digestion optimization**

*Areas of native (light, AreaL) and TQL (heavy, AreaH) peptides after 8, 16 and 24 h trypsin digestion. 16 h trypsin digestion was determined as optimal based on the highest yield.*

**Supplemental Figure 3: Correlation between ADRP peptides**

**
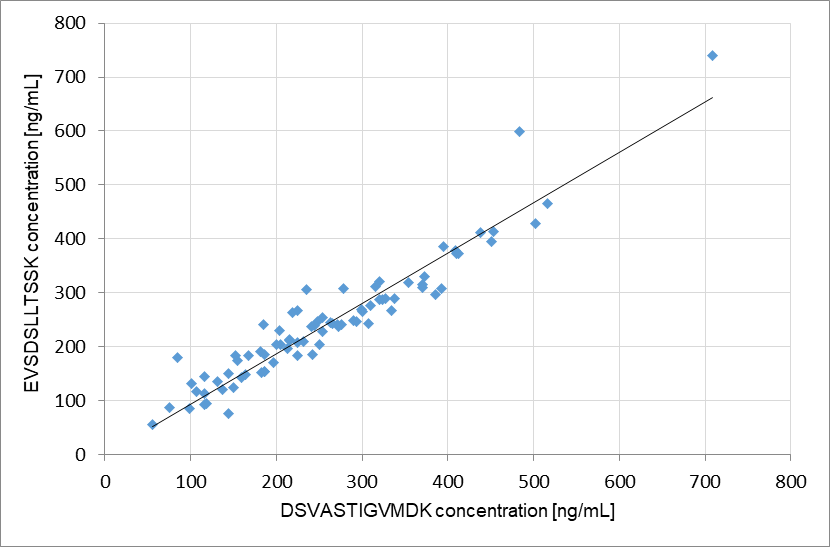
**

*The correlation between peptides DSVASTIGVMDK and EVSDSLLTSSK in breast milk samples. These peptides were used for ADRP quantification, their mean concentration was determined as final ADRP concentration.*

**Supplemental Figure 4: Correlation between ADRP, TIP47 and total lipid content visualized using PCA**

**
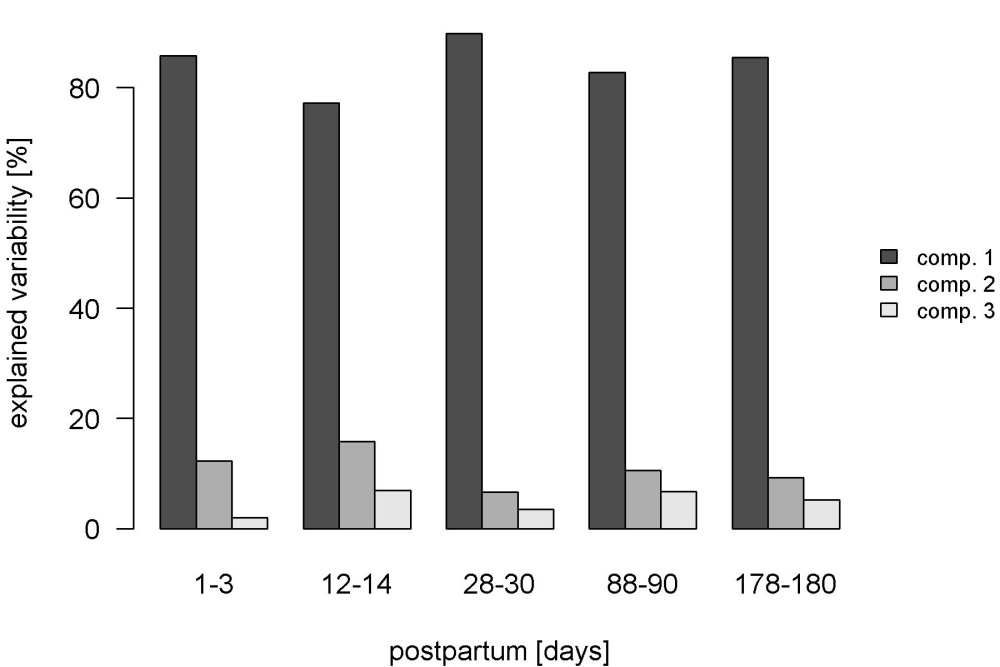
**

Principal component analysis enables to uncover correlation structure within a set of variables, in this case ADRP, TIP47 and total lipid concentrations. First component contains around 80 % of variability of the three variables in each time point, suggesting close correlation between the three variables.
